# Exosome-Based Therapeutics in Alzheimer’s Disease: Translational Perspectives Beyond Conventional Therapies

**DOI:** 10.3390/cimb48080808

**Published:** 2026-08-11

**Authors:** Eleni G. Andreadou, Evangelia Evangelopoulou, Magda Tsolaki, Ioannis Tsamesidis, Ilias Pessach, Stella Mitka

**Affiliations:** 1 Department of Biomedical Sciences, International Hellenic University, Sindos, 57400 Thessaloniki, Greece; liaeuag2016@gmail.com (E.E.); itsamesidis@auth.gr (I.T.); iliaspessach1980@gmail.com (I.P.); mitka@ihu.gr (S.M.); 2 Laboratory of Neurodegenerative Diseases, Center for Interdisciplinary Research and Innovation (CIRI), Aristotle University of Thessaloniki, Thermi, 57001 Thessaloniki, Greece; tsolakim1@gmail.com; 3 Greek Association of Alzheimer’s Disease and Related Disorders (GAADRD), 54643 Thessaloniki, Greece; 4 1st Department of Neurology, Faculty of Medicine, Aristotle University of Thessaloniki, 54124 Thessaloniki, Greece

**Keywords:** Alzheimer’s disease, exosomes, extracellular vesicles, monoclonal antibodies, drug delivery, blood–brain barrier, precision medicine, translational medicine

## Abstract

Alzheimer’s disease (AD) remains a major neurodegenerative disorder lacking effective long-term disease-modifying therapies. Current pharmacological approaches provide primarily symptomatic benefit, while recently approved anti-amyloid monoclonal antibodies offer only modest clinical efficacy and are constrained by safety concerns, high costs, and limited blood–brain barrier (BBB) penetration. In this context, exosome-based strategies have emerged as potential therapeutic platforms in AD research. As endogenous nanovesicles, exosomes exhibit favorable biocompatibility, intrinsic BBB-crossing capacity, and the ability to deliver diverse therapeutic cargo across multiple pathological pathways. Through bioengineering approaches, exosomes may additionally be optimized for targeted brain delivery and therapeutic personalization. Nevertheless, despite their conceptual and preclinical advantages, exosome-based therapies remain at an early translational stage, with unresolved challenges related to large-scale production, standardization, biodistribution, and long-term safety. This review provides a comparative translational analysis of conventional and exosome-based therapeutic strategies in AD, focusing on mechanistic targeting, delivery efficiency, safety and tolerability, therapeutic personalization, and translational readiness. We critically evaluate the extent to which exosome-based platforms may address the limitations of current therapies while highlighting the key barriers that continue to limit their clinical translation and real-world applicability.

## 1. Introduction

As the leading cause of dementia worldwide, Alzheimer’s disease (AD) affects nearly 50 million people, with its prevalence mainly concentrated in the elderly population [1,2]. The clinical manifestations of AD arise from a complex and multifactorial pathology. AD pathogenesis is primarily associated with two key features: the extracellular accumulation of β-amyloid (Aβ) plaques and the formation of intracellular neurofibrillary tangles composed of hyperphosphorylated tau protein. This toxic environment is further exacerbated by neuroinflammation, oxidative stress, and mitochondrial dysfunction. These neurodegenerative processes begin years before overt cognitive decline, during a silent prodromal phase, presenting a critical yet challenging window for therapeutic intervention [3].

Despite recent therapeutic advances, no definitive cure for AD currently exists. The available therapeutic strategies primarily aim to provide symptomatic relief and modest delay of disease progression [4]. Cognitive symptoms are primarily managed with acetylcholinesterase inhibitors (donepezil, rivastigmine, galantamine) and the N-methyl-D-aspartate receptor antagonist memantine. These drugs are approved by the Food and Drug Administration (FDA) [5]. Nonetheless, these are short-term interventions, as they fail to address the core pathogenetic mechanisms and are associated with adverse effects [5]. The recent approval of anti-Aβ monoclonal antibodies such as aducanumab, lecanemab and donanemab represents a shift toward disease-modifying therapies [6]. However, their clinical benefit remains modest and restricted to the earlier stages of AD, while safety concerns, including amyloid-related imaging abnormalities (ARIA), necessitate close monitoring [5,7]. These immunotherapies are also associated with substantial costs [5], while their limited ability to cross the blood–brain barrier (BBB) further restricts therapeutic delivery [8].

As the biological complexity of AD continues to challenge therapeutic development [9], the discovery of effective treatments is urgent [10]. An additional obstacle is the heterogeneity of disease phenotypes, which, combined with diagnostic uncertainty arising from co-existing pathologies and overlapping neurodegenerative processes, complicates patient stratification and therapeutic selection. These factors render precision medicine a promising yet challenging therapeutic strategy [11].

In the field of nanomedicine, synthetic nanocarriers such as liposomes, metallic and polymeric nanoparticles, and dendrimers have been developed for the treatment of various diseases [12]. Nevertheless, several synthetic nanocarriers have been reported to face issues regarding biocompatibility, immunogenicity, toxicity, or BBB penetration, depending on their composition and formulation [13]. In this context, small extracellular vesicles (EVs) have emerged as an alternative nanoplatform for brain drug delivery [14]. Exosomes are nanovesicles (30–150 nm) secreted by almost all cell types. They play an important role in intercellular communication by transferring molecular cargo (e.g., proteins, lipids, and nucleic acids) [15,16].

Their endogenous origin confers key advantages, including intrinsic biocompatibility, prolonged circulation time, low immunogenicity, inherent tissue tropism, and the ability to cross the BBB [13,17]. More specifically, exosomes are being explored as delivery vehicles capable of overcoming pharmacokinetic limitations and BBB restrictions [18]. They can transport proteins, nucleic acids, and drugs, exerting neuroprotective and anti-inflammatory effects [19]. Moreover, they can be engineered through cargo loading or surface modification to enable targeted delivery, supporting their development as cell-free therapeutic platforms [20,21].

Despite growing interest in exosome-based therapeutics, comprehensive translational comparisons with conventional therapeutic approaches remain scarce. Consequently, the extent to which exosome-based platforms can realistically address the limitations of current therapies and support more personalized treatments in AD has not yet been clarified. This review provides a comparative translational assessment of conventional and exosome-based therapeutic strategies in AD, focusing on mechanistic targeting, delivery efficiency and BBB penetration, safety and tolerability, therapeutic personalization, and translational readiness.

## 2. Materials and Methods

This review was designed as a qualitative narrative synthesis to critically compare conventional treatments with emerging exosome-based delivery systems in AD. The analysis focuses on bridging the gap between established therapeutic modalities and next-generation platforms, evaluating their mechanistic alignment with disease complexity and their potential for clinical translation.

To achieve this objective, a comprehensive literature search was conducted across PubMed, Scopus, and Google Scholar databases using Boolean operators and keywords, including (“Alzheimer’s disease” OR “AD”) AND (“therapeutics” OR “drug delivery”) AND (“exosomes” OR “engineered exosomes”) AND (“monoclonal antibodies” OR “targeted delivery”). The search focused on English-language publications from 2015 to 2026, with special emphasis on high-impact longitudinal studies and clinical trial reports from 2020 onwards.

The selection process prioritized studies reflecting the comparative therapeutic scope of this review. Specifically, the inclusion criteria prioritized clinical evidence from randomized controlled trials and post-marketing surveillance of FDA-approved drugs, as well as technological innovations in exosome engineering, cargo loading, and targeting specificity. Furthermore, the selection accounted for pharmacokinetic data regarding BBB permeability and central nervous system (CNS) bioavailability, while also incorporating regulatory guidelines from agencies such as the FDA to ensure clinical relevance.

The selected literature was subsequently synthesized into a structured multidimensional framework, and each strategy was assessed across five critical domains. First, mechanistic targeting was evaluated based on the alignment of molecular targets with the multifactorial pathophysiology of AD. Second, delivery efficiency was analyzed in terms of CNS delivery and the dose-exposure relationship in the brain. Third, safety and tolerability profiles were examined, focusing on immunogenic potential, long-term toxicity, and monitoring requirements. Additionally, the review assessed the capacity for therapeutic personalization, including the integration of genetic profiling, biomarkers, and AI-driven stratification. Finally, translational readiness was evaluated based on regulatory maturity, manufacturing scalability, and the feasibility of cost-effective integration into modern healthcare systems.

## 3. Results

The therapeutic landscape in AD continues to evolve, with ongoing efforts to develop strategies that modify disease progression while overcoming the limitations of currently available treatments. Despite these advances, the overall outlook for patients with AD is poor in the absence of a definitive cure, underscoring the need for continued therapeutic innovation and comparative evaluation of emerging therapeutic strategies [1,4,5,6,7,8].

Accordingly, this review comparatively evaluates conventional pharmacotherapies, anti-amyloid monoclonal antibodies (mAbs), and exosome-based therapeutic strategies across five clinically relevant parameters to better define their respective advantages, limitations, and translational potential. The overall comparative framework is presented in Section 3.2.

### 3.1. Research Background

Acetylcholinesterase inhibitors (AChEIs) and the partial N-methyl-D-aspartate (NMDA) receptor antagonist memantine appear to improve executive and cognitive function, as well as activities of daily living in patients with AD [5]. Although these treatments cannot reverse the neurodegenerative process, they may slow disease progression for up to 5 years by contributing to symptomatic relief [5,22]. Monotherapy with memantine has been associated with improvements in cognition, behavioral symptoms, activities of daily living, and overall clinical condition, while combination therapy with donepezil may enhance therapeutic response [5,23]. However, due to adverse effects and interpatient variability in therapeutic response, regular clinical monitoring is required to assess treatment efficacy and tolerability [5].

More recently, second-generation anti-Aβ mAbs, including aducanumab, lecanemab and donanemab, have introduced disease-modifying therapies by selectively targeting toxic Aβ aggregates [24]. Among these therapies, recent developments with donanemab have further expanded treatment strategies through biomarker-guided therapeutic approaches [25,26]. Despite representing an important transition toward disease-modifying therapies, their clinical application is constrained by safety concerns and limited BBB penetration, necessitating high systemic exposure that may increase toxicity risk and adverse reactions [8,27].

In parallel with advances in conventional pharmacotherapy, exosomes have emerged as promising therapeutic carriers owing to their favorable biological properties, including biocompatibility, prolonged circulation, and targeted delivery capability. Their ability to cross the BBB further supports their potential for CNS drug delivery [17]. Exosome-based therapeutic strategies can be broadly classified into native exosomes and engineered exosomes. Engineered exosomes are modified to enhance their therapeutic performance [19]. Bioengineering strategies, including cargo loading with bioactive molecules (e.g., drugs, RNAs or peptides) and surface modification, are used to enhance targeting efficiency, stability and immune evasion [19,20,21]. Interaction with target cells is mediated by exosomal surface markers that facilitate selective uptake by neuronal and glial populations [28].

Native exosomes exert therapeutic effects through their endogenous bioactive cargo. For instance, neuron-derived EVs enriched in miR-124-3p appear to enhance astrocytic glutamate uptake, thereby attenuating excitotoxicity [29]. Astrocyte-derived exosomes containing high levels of apolipoprotein D promote neuronal survival under oxidative stress conditions [30]. In addition, neuron-derived EVs have been shown to bind Aβ and facilitate its microglial uptake and lysosomal degradation, potentially contributing to endogenous amyloid clearance mechanisms [31].

Engineered exosomes are bioengineered EVs that are modified through genetic, chemical, or physical approaches to enhance targeted therapeutic delivery. For instance, exosomes modified with the Rabies Virus Glycoprotein (RVG) peptide have been used to deliver short interfering RNA (siRNA) targeting the BACE1 gene, resulting in suppression of amyloid burden in AD models [32,33]. Engineered exosomes have been explored as delivery systems for gene-editing technologies such as CRISPR-Cas9 [34,35], as well as carriers for natural compounds and conventional drugs, including curcumin, quercetin, donepezil and aducanumab, with studies reporting improved BBB penetration, enhanced bioavailability and reduced immune-mediated clearance in experimental AD models [35,36,37,38,39,40,41]. Surface and lipid modifications additionally enhance exosomal stability, circulation time and tissue specificity [13,19,42].

Among the different exosome sources, mesenchymal stem cell-derived exosomes (MSC-Exos) have attracted particular interest owing to their favorable biocompatibility and safety profile [37]. As a cell-free alternative to stem cell transplantation, MSC-Exos retain several beneficial properties of their parent cells while avoiding some limitations associated with direct cell therapy, such as oncogenesis and immune rejection [17,43,44]. They also contain enzymes involved in Aβ degradation, including neprilysin and insulin-degrading enzyme, which may contribute to the clearance of pathological aggregates [36,37]. Experimental studies also suggest that hypoxia-preconditioned MSC-Exos reduce Aβ accumulation and suppress neuroinflammation, while promoting the shift of hippocampal microglia from the pro-inflammatory (M1) to the anti-inflammatory (M2) phenotype in AD mouse models [43,44,45].

To date, a Phase I/II clinical trial (NCT04388982) has evaluated intranasally administered allogeneic adipose-derived MSC-Exos in patients with mild-to-moderate AD [46,47]. The treatment was well tolerated and associated with improvements in cognitive scores; however, these findings should be interpreted cautiously owing to the small sample size and incomplete mechanistic understanding [47,48].

Despite these encouraging findings, the clinical translation of exosome-based therapies still faces several challenges, including incomplete understanding of pharmacokinetics, long-term safety, potential immune responses to engineered exosomes, and lack of standardized large-scale production and quality control protocols [14,19,30,49]. Additionally, variability related to MSC source selection, isolation methods, and dosing strategies continues to complicate the reproducibility of therapeutic outcomes, highlighting the need for rigorous large-scale clinical validation [14,30].

### 3.2. Comparative Evaluation of Conventional and Exosome-Based AD Therapeutic Strategies

#### 3.2.1. Mechanistic Targeting

Knowledge surrounding the pathophysiology of AD has expanded substantially in recent years, driven by advances in molecular neuroscience and systems pharmacology [50]. Multiple hypotheses, including the amyloid, tau, and cholinergic models, have been proposed. Despite that, each hypothesis applies to only a subset of patients or explains only part of the disease process [51]. AD is now widely recognized as a multifactorial, network-driven disorder that cannot be attributed solely to amyloid-centric or tau-centric pathology [50,52]. This concept is supported by the observation that sporadic AD, which accounts for at least 95% of cases, arises from multiple etiologies and interacting mechanisms rather than a single initiating trigger [51]. Accordingly, hallmark pathological features interact within a complex cascade, exacerbating neuronal loss and cognitive decline [50,53].

This intricate molecular interplay, which underlies the multidimensional nature of AD, renders single-target therapeutic approaches insufficient for effective disease management [50,52]. The repeated failure of pharmacological agents that modulate a single pathogenic pathway may therefore be partially explained by their limited efficacy across the broader patient population, as they often benefit only a small subgroup [51]. Consequently, multi-target therapeutic strategies that simultaneously address interconnected pathological mechanisms (e.g., protein aggregation, synaptic dysfunction, neuroinflammation) have drawn increased attention [50,51,52].

Most monotherapeutic strategies dominating recent research efforts act by inhibiting amyloid plaque formation, suppressing cholinesterase activity, or targeting pathways involved in tau hyperphosphorylation [52]. The primary therapeutic objective is to improve cognition and memory by modulating neuronal factors implicated in disease progression [53].

At present, cholinergic and glutamatergic systems constitute the two main pharmacological targets in AD therapy, corresponding to acetylcholine and glutamate, neurotransmitters that play fundamental roles in learning and memory [54]. Approved conventional pharmacotherapies, AChEIs and NMDA receptor antagonists, primarily address cognitive and behavioral symptoms rather than the underlying neurodegenerative process, with their modest efficacy typically confined to specific patient subgroups [50,54].

Disease-modifying therapies, most notably mAbs, are also single-target interventions, primarily aimed at enhancing the clearance of accumulated Aβ and thereby slowing cognitive decline. Amyloid-targeting therapies partially modulate systemic molecular and cellular disturbances; however, their overall impact remains restricted [50]. According to the 2023 report of the National Institute on Aging and the Alzheimer’s Association, most agents currently in the development pipeline fall within the disease-modifying category. Among these, anti-amyloid and anti-inflammatory agents constitute the dominant classes, while the remaining candidates target tau pathology, mitochondrial dysfunction, and synaptic impairment [52].

Despite this progress, complex diseases such as AD inherently require multifocal therapeutic strategies, as modulation of a single pathway may trigger compensatory responses within interconnected biological networks. Moreover, several pathological processes exhibit dynamic behavior, shifting from neuroprotective roles at early disease stages to neurotoxic effects later in progression, further complicating the identification of optimal therapeutic windows [50]. Therefore, effective treatment development requires a comprehensive understanding of the pathways involved in disease onset and progression, as well as their complex interrelationships, even though the precise initiating mechanisms of AD are still under investigation [52,53].

Although multi-target drug development strategies for AD have gained increasing attention in recent years, their broader adoption remains incomplete. This may be attributed to the entrenched single-target nature of FDA-approved therapies, their long-standing clinical use, and the inherent complexity of evaluating multi-mechanistic drug actions. Nevertheless, overcoming these limitations and embracing non-conventional therapeutic strategies is increasingly regarded as essential for meaningful progress in AD drug development [51].

In contrast to conventional single-target therapeutic approaches, exosomes are explored as potential multi-target therapeutic platforms, capable of simultaneously targeting multiple pathological pathways [55]. Their capacity for surface modification with brain-targeting peptides, combined with their ability to encapsulate diverse therapeutic cargos—such as BACE1-targeting siRNA, anti-inflammatory microRNAs and neurotrophic factors—supports their potential as adaptable platforms for targeted therapy rather than simple natural nanocarriers [55,56].

Notably, engineered exosomes integrate advantageous features of both synthetic nanocarriers and cell-based therapies [55]. Beyond their ability to cross the BBB, their multi-target functionality influences Aβ clearance, synaptic integrity, and anti-inflammatory activity [57]. Through rational engineering that enables brain targeting and stimulus-responsive release of therapeutic cargo, engineered exosomes could simultaneously facilitate the clearance of amyloid plaques and neurofibrillary tangles while suppressing neuroinflammation, thereby delineating a multidimensional therapeutic option for neurodegenerative diseases [55].

The principal advantage of exosomes over single-target therapies lies in their native biology, including their biogenesis and structural adaptability [55]. Specifically, during exosome biogenesis, therapeutic molecules can be incorporated beyond endogenous cargo, enabling multi-focal therapeutic activity [58]. In addition, proteins mediating cellular interactions, including tetraspanins and integrins, are naturally enriched in the exosomal lipid bilayer [55]. Collectively, the presence of a modifiable outer membrane and a high-capacity internal lumen allows exosomes to act simultaneously on specific cell populations addressing distinct pathological pathways [59]. Nevertheless, exosomes may also contribute to AD pathophysiology, including the spreading of Aβ, indicating that their role is context-dependent and requires careful consideration in therapeutic design [36].

Taken together, engineered exosomes appear to surpass conventional therapeutic approaches in mechanistic pathway targeting by enabling simultaneous intervention across multiple core pathological pathways of AD while constituting an integrated therapeutic platform. However, this advantage depends on rational design and appropriate exploitation of exosomal properties. Their biological role is context-dependent owing to their potential involvement in both neuroprotection and the propagation of pathological proteins.

#### 3.2.2. Delivery Efficiency and BBB Penetration

The pursuit of optimal drug bioavailability within the CNS remains a major challenge due to the semi-permeable nature of the BBB, which constrains targeted delivery to neurodegenerative diseases [18,60,61]. Consequently, higher systemic doses are often required to achieve the minimum effective concentration in the brain [60]. The inadequate CNS accumulation markedly limits the efficacy of conventional therapies, as the BBB is impermeable to macromolecules and approximately 98% of small-molecule compounds [53,61].

Small lipophilic molecules can cross the BBB via distinct transport mechanisms, with passive diffusion favoring low-molecular compounds with appropriate physicochemical properties [18,61]. Nevertheless, most strategies designed to enhance BBB permeability have either compromised barrier integrity or failed to demonstrate clinically meaningful efficacy [18].

Conventional therapies are administered primarily orally as tablets or capsules [53]. Rivastigmine is also available as a transdermal patch for sustained drug release and has demonstrated clinical efficacy in patients with AD [62]. Although these agents can cross the BBB via passive transcellular diffusion [61], their therapeutic efficiency remains limited by first-pass metabolism, plasma protein binding, systemic distribution, and peripheral adverse effects [53]. For instance, memantine undergoes gastrointestinal (GI) absorption and hepatic metabolism with substantial renal excretion, whereas donepezil exhibits extensive plasma protein binding and suboptimal final BBB penetration (15.7% of total drug). Therefore, pharmacokinetic parameters (absorption, distribution, metabolism, and clearance) require optimization, as increased systemic exposure is frequently associated with higher toxicity risks [53].

Compared with conventional pharmacotherapies, mAb therapies highlight the limitations of CNS delivery, as only approximately 0.1% of the intravenously administered dose crosses the BBB. Although dose escalation is associated with increased Aβ clearance, clinical benefit remains modest and is accompanied by greater safety concerns [8]. These limitations have highlighted targeted brain delivery as a prerequisite for therapeutic efficacy and stimulated the investigation of alternative delivery systems which incorporate conventional therapeutics into vesicular systems [53].

To overcome these obstacles, research has focused on developing nanocarriers which can cross the BBB while delivering active pharmaceutical agents with minimal toxicity [60]. Unlike conventional drug formulations, exosomes express surface receptors such as transferrin and insulin receptors, as well as integrins, allowing them to interact with BBB endothelial cells and cross the barrier primarily via receptor-mediated transcytosis. Fusion and paracytosis mechanisms have also been proposed. Notably, brain-derived exosomes exhibit intrinsic tropism toward neural cells and participate in intercellular communication within the neurovascular unit, thereby enhancing CNS-targeted delivery [18].

Furthermore, exosomes can undergo surface engineering to improve cell-specific targeting, as exemplified by conjugation of the RVG peptide to Lamp2b for neuronal targeting through acetylcholine receptors [60,63], while neuropilin-binding peptides and neural nanobody fusions are also being investigated to enhance CNS homing [63].

Importantly, MSC-Exos have demonstrated preferential accumulation in regions of neuropathology, particularly within neuronal populations, suggesting that neuroinflammatory signaling may contribute to homing mechanisms. In AD mouse models, intranasal administration resulted in enhanced targeting of the hippocampus [18], although homing efficiency compared to their parent MSCs is lower and may affect their overall therapeutic efficacy [64].

**Figure 1:**
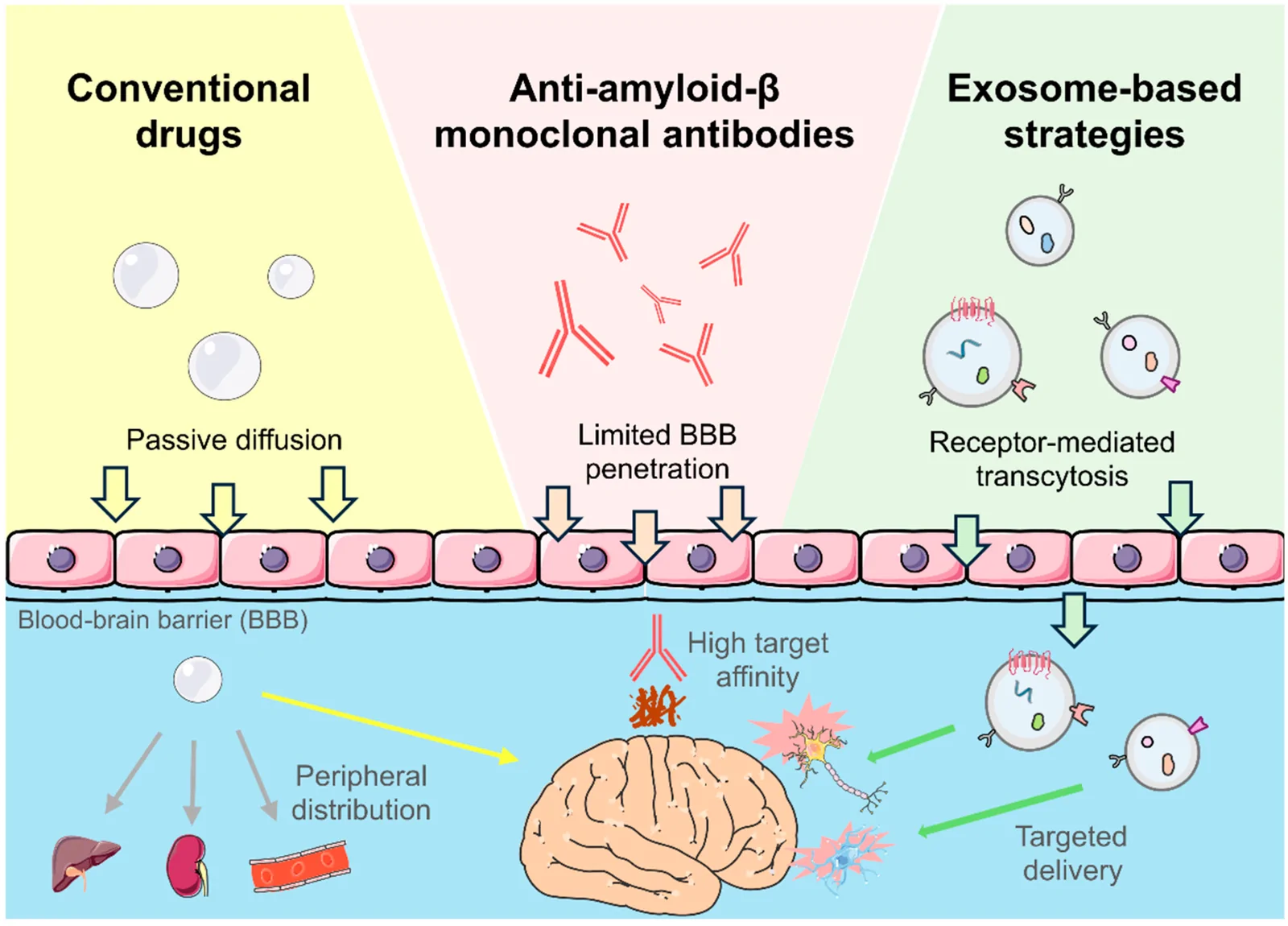
Comparative overview of blood–brain barrier (BBB) penetration and central nervous system (CNS) delivery characteristics of conventional drugs, anti-Aβ monoclonal antibodies (mAbs), and exosome-based strategies for Alzheimer’s disease therapy. Conventional drugs primarily rely on passive diffusion and exhibit broad peripheral distribution. Anti-Aβ mAbs demonstrate high target affinity but limited BBB penetration following intravenous administration. Exosome-based strategies exploit endogenous transport mechanisms, including receptor-mediated transcytosis, to enhance targeted CNS delivery of therapeutic cargo.

Targeted biodistribution may also reduce off-target uptake by peripheral organs such as the liver and spleen while prolonging systemic circulation [63]. Expression of CD47 on exosomes prevents macrophage-mediated clearance via the “don’t eat me” signaling pathway [18,63]. Optimization of vesicle size and surface charge may minimize renal clearance and nonspecific uptake, provided that intrinsic physicochemical properties are preserved [63]. The principal differences in BBB penetration and CNS delivery among conventional drugs, anti-Aβ mAbs, and exosome-based strategies are summarized in Figure 1.

Compared with currently available routes of administration for conventional therapies, intranasal delivery has emerged as a particularly attractive strategy for brain targeting, as it bypasses first-pass metabolism and exploits the neural and vascular connectivity of the nasal epithelium to facilitate direct CNS access. Although reduced absorption and rapid mucociliary clearance remain limitations, these challenges may be partially mitigated through permeability enhancers and mucoadhesive excipients [53].

As a final point, important uncertainties regarding exosome-based delivery systems, including in vivo biodistribution, long-term accumulation, and precise BBB transport, should be acknowledged [60]. Moreover, the mechanistic involvement of exosomes in AD pathology remains only partially elucidated and necessitates further investigation [18,60].

Collectively, efficient BBB penetration and targeted CNS biodistribution constitute critical determinants of therapeutic success. Conventional drugs cannot target specific areas efficiently. Although they partially cross the BBB, they distribute nonspecifically and act only symptomatically, resulting in low accumulation at the target site. Similarly, mAbs exhibit extremely low CNS bioavailability despite high target affinity, necessitating dosing strategies that may increase toxicity risks. Exosomes may partially overcome these limitations through endogenous transport mechanisms and enhanced targeting capacity, thereby representing a promising delivery platform for AD therapeutics. Nevertheless, subsequent studies are required to better understand their in vivo biodistribution, long-term fate, and transport mechanisms across the BBB.

#### 3.2.3. Safety and Tolerability

The “one target-one drug” paradigm, adopted in drug development to avoid off-target interactions and associated adverse effects (AEs), has demonstrated limited effectiveness and, in many cases, unfavorable safety profiles. The complexity and extensive crosstalk of biological networks may further contribute to unintended off-target effects and reduced therapeutic tolerability [65]. Safety and tolerability are, therefore, critical parameters for evaluating therapeutic effectiveness, particularly in the context of polypharmacy, AEs, and frequently reduced patient adherence [53].

Regarding symptomatic therapies, AChEIs are primarily associated with GI-related side effects (5–20%), sleep disorders, and bradycardia, thereby limiting tolerability and treatment adherence [23,54]. Interactions with other anticholinergic drugs (e.g., antidepressants, antihistamines) may result in additional AEs and cognitive impairment [54]. Nevertheless, these side effects are generally considered tolerable in the long term, and AChEIs remain the mainstay treatment for mild AD [66]. NMDA receptor antagonists are also associated with AEs that may compromise tolerability and adherence, including dizziness, headache, and agitation. Similarly, interactions with pharmacologically related agents may increase this risk [54]. Even so, their safety profile is considered relatively predictable, and memantine is generally well tolerated following oral administration [53].

Despite these generally manageable safety profiles, anti-amyloid mAb therapies are primarily associated with ARIA, which constitutes their principal safety concern. Parenchymal hemorrhage may induce neurological symptoms, particularly at higher doses in the context of ARIA with edema (ARIA-E), and may rarely result in fatal outcomes [53,54,67]. ARIA subtypes are detected via Magnetic Resonance Imaging (MRI) monitoring [54], while intravenous administration every 2–4 weeks further increases the clinical and logistical burden associated with treatment implementation [53,66]. In addition, ARIA has been associated with brain atrophy, raising additional concerns regarding the long-term safety profile of mAb therapies despite their therapeutic efficacy [53]. The occurrence of symptomatic ARIA may necessitate dose adjustment or treatment discontinuation [54], whereas ARIA with hemorrhage (ARIA-H) is typically asymptomatic [67].

Apolipoprotein E (ApoE) ε4 carriers, a risk group for AD, exhibit significantly higher incidence rates for ARIA [54,66,67]; therefore, ApoE genotyping is recommended in clinical practice [6]. Other safety concerns include autoimmune reactions (e.g., allergies, inflammation, infections, injection site reactions) and insufficient BBB penetration [54], with higher therapeutic doses increasing toxicity risk and treatment-related AEs [27].

Regarding lecanemab and donanemab, their clinical benefit remains modest, while safety concerns are significant, including FDA warnings about potentially fatal complications in high-risk patients. Nonetheless, these agents are still therapeutic options for Mild Cognitive Impairment (MCI) [66]. These mAbs also differ in their mechanisms of action and ARIA risk profiles [66], with reported incidence rates of 12.6% and 24% for ARIA-E, and 17.3% and 19.7% for ARIA-H, respectively [8,68]. Network meta-analysis data in MCI/mild AD patients indicate that aducanumab showed the highest efficacy based on cognitive scores but also the greatest ARIA risk. On the other hand, donanemab showed comparatively lower efficacy [67]. Notably, aducanumab was discontinued by Biogen in January 2024 following strategic reprioritization toward lecanemab and economic considerations rather than explicit safety or efficacy [69].

Post-marketing pharmacovigilance data from the FDA Adverse Event Reporting System have additionally reported unexpected AEs associated with lecanemab, including migraines, tremors, and pancreatic cancer. Patients receiving polypharmacy or concomitant medications (e.g., aspirin, statins, antidepressants) appear to be at greater risk of severe complications [67]. Taken together, these findings underscore the need for individualized administration, strict longitudinal monitoring, and cautious patient selection when applying anti-Aβ mAb therapies [66,67]. Their long-term safety profile is also under evaluation through ongoing post-marketing surveillance studies [66].

Exosomes have shown low immunogenicity as natural drug delivery vehicles, while their favorable biocompatibility and prolonged circulation half-life further support their capability for safer therapeutic delivery [70]. Compared with their parent cells, MSC-Exos demonstrate lower immunogenicity and improved tolerability. The only completed clinical trial to date (NCT04388982), involving allogeneic adipose-derived MSC-Exos, reported favorable safety and tolerability, with no serious adverse events observed during the 12-week treatment period and no detectable toxicity in vital organs such as the liver and kidneys [71].

Despite these encouraging findings, several safety considerations regarding exosome-based therapeutics persist. Long-term toxicity, immune activation, and off-target accumulation have not yet been fully elucidated [71]. Following systemic administration, exosomes may undergo nonspecific uptake by the mononuclear phagocyte system, particularly in the liver and spleen, potentially contributing to off-target accumulation and organ toxicity [55]. Although allogeneic exosomes are generally considered to have a favorable immunological profile, their surface proteins, lipid composition, or engineered modifications may still trigger immune activation, inflammatory responses, or anti-drug antibody formation following repeated administration [12,30,55,72].

The biological role of endogenous exosomes in AD is complex, as neuron-derived EVs have been shown to carry Aβ and hyperphosphorylated tau and may contribute to their intercellular propagation under specific pathological conditions. Conversely, endogenous EVs have also been implicated in Aβ sequestration, microglial clearance, and other neuroprotective mechanisms, indicating that their effects may vary according to cellular origin and disease context [36]. Although MSC-Exos generally exhibit low immunogenicity, harmful paracrine signaling or insufficient purification may still hamper their therapeutic efficacy and lead to co-transfer of pathogenic cargo [55,71]. Therefore, the dual biological role of endogenous EVs highlights the need for comprehensive long-term safety assessment of therapeutic MSC-Exos before broader therapeutic application [36,71].

Overall, the three therapeutic platforms exhibit distinct safety profiles that largely reflect their respective mechanisms of action and stage of clinical development. Symptomatic therapies exhibit a relatively predictable and clinically established safety profile based on extensive long-term use, despite clear limitations in tolerability. Safety-related considerations that currently hinder the clinical applicability of mAbs include ARIA-related risks, intensive MRI monitoring requirements, and the increased toxicity associated with systemic dose escalation. Accordingly, careful patient selection and individualized risk-benefit assessment are regarded as essential. Exosomes represent a potentially safer therapeutic platform, supported primarily by encouraging preclinical evidence and the absence of serious AEs reported thus far. However, they are still at an early clinical validation stage for definitive safety evaluation. Although their high biocompatibility and favorable tolerability are promising, long-term safety evaluation remains necessary before their clinical value can be fully established.

#### 3.2.4. Therapeutic Personalization

AD exhibits significant heterogeneity across multiple levels, including etiology, pathophysiology, clinical manifestations, genetic background, and patient lifestyle factors [51,73]. Personalized or Precision Medicine (PM) aims to tailor treatment to each patient’s susceptibility and predicted therapeutic response while adopting the paradigm of “the right treatment for the right patient at the right time” [74,75]. Personalization encompasses tools such as biomarkers (fluid, imaging, digital), genetic profiling (e.g., ApoE ε4), multi-omics data, and AI-based predictive models [50,66,76].

Among the currently available therapies, only a subset of patients appears to derive meaningful benefit [50]. Clinical experience has consistently shown a considerable inter-patient variability in treatment response [50], likely reflecting the limitations of the prevailing “one size fits all” strategy [77]. In addition, a considerable proportion of patients experience AEs, drug–drug interactions, poor tolerability, and reduced treatment compliance [78]. This heterogeneity in therapeutic response supports the development of personalized approaches guided by biomarker information, genetic profiles, and individual risk factors [50,77,78]. The primary aim of PM is to optimize prevention and treatment strategies to maximize efficacy, minimize AEs, and support accurate disease diagnosis and monitoring [77].

Symptomatic pharmacological treatments are inherently non-personalized, as they function independently of underlying pathogenic mechanisms and aim to provide temporary symptomatic relief [50]. Dose adjustment and treatment duration are modified according to clinical response and tolerability, representing only a limited form of individualized treatment management rather than true mechanistic personalization [79]. In the case of AChEIs, gradual dose titration is applied according to patient tolerance and therapeutic response [78,80]. Memantine follows a similar “start low, go slow” strategy with supplementary adjustments for vulnerable populations such as patients with renal impairment [81]. Moreover, AChEIs are approved for mild-to-moderate AD, whereas NMDA receptor antagonists and combination therapies are indicated for moderate-to-severe disease stages. This classification is based primarily on efficacy criteria rather than individualized targeting of pathogenic mechanisms [5,62].

Compared with symptomatic pharmacological treatments, mAb therapies are currently administered only to patients with MCI or mild AD and exert their effects through fixed mechanisms that target specific forms of Aβ and promote its degradation via microglial cells [79]. Although their use requires confirmation of amyloid pathology via Positron Emission Tomography (PET) or cerebrospinal fluid (CSF) biomarkers, as well as MRI monitoring [79], these therapies do not constitute genuinely personalized interventions. ApoE genotyping and strict patient selection criteria are mainly applied for eligibility assessment and risk stratification purposes [6,50,79]. Treatment modifications largely involve cautious dose escalation and monitoring driven by safety considerations rather than individualized therapeutic optimization [54,79].

PM also incorporates advanced tools such as genomics, metabolomics, transcriptomics, and AI-based analytical models that could integrate multimodal datasets to identify predictive biomarkers, potential drug interactions, and patient-specific molecular profiles [50,76]. In conventional therapeutic frameworks, these tools may support risk stratification [66], pre-symptomatic assessment, longitudinal monitoring via Electronic Health Records [74], and biomarker-guided patient selection according to molecular and demographic characteristics [73,77].

Beyond the personalization strategies theoretically applicable to conventional therapeutic approaches, exosome-based systems may offer broader opportunities for therapeutic personalization, provided that the mechanisms underlying their biological effects are sufficiently understood [64]. Specifically, exosomes enable the selection of their cellular origin [19], the incorporation of tailored bioactive cargo, and the modification of their surface markers to improve targeting efficiency and reduce off-target effects [13,21]. MSC-Exos additionally retain several beneficial properties of their parent cells [44]. Their inherent biological characteristics, combined with the possibility of laboratory engineering for patient-specific applications, support their potential role as adaptable therapeutic platforms, particularly in cell-free regenerative medicine [21]. Furthermore, integrating therapeutic exosomes with exosome-derived biomarkers may contribute to the development of theranostic frameworks that support clinical decision-making and real-time treatment monitoring [20,82].

The integration of multi-omic datasets with AI-based analytical tools may promote the development of models that reflect the multidimensional nature of AD and facilitate mechanism-based therapeutic strategies [50,75]. In the context of exosome-based therapies, these methods could potentially guide cargo selection, therapeutic target identification, and intervention timing, thereby promoting a transition toward patient-centered therapeutic design [50,76]. In such settings, personalization extends beyond patient stratification to shaping the therapeutic platform itself [73,75,76].

Despite this conceptual advantage, personalized exosome-based therapies remain largely confined to the preclinical stage due to unresolved challenges regarding dosage optimization, administration strategies, and incomplete understanding of their mechanisms of action [64]. More broadly, implementation of PM in AD is currently hindered by disease heterogeneity, biomarker instability, data integration complexity, and the need for robust validation of predictive models across diverse patient populations [50,76,77].

In summary, symptomatic therapies largely follow fixed regimens, with personalization primarily constrained to dose adjustment and tolerability management. Monoclonal antibody therapies require biomarker-based patient selection; however, this approach mainly supports eligibility assessment and risk stratification. In conventional therapeutic strategies, PM tools currently demonstrate greater potential to improve patient stratification and support earlier intervention strategies than to enable true therapeutic personalization. In contrast, exosome-based platforms show substantial conceptual and preclinical value for therapeutic personalization, although further clinical evaluation is required. In the long term, integration of PM tools with exosome-based therapeutic platforms may support the development of theranostic and patient-centered intervention strategies in AD.

#### 3.2.5. Translational Readiness

Despite considerable progress over the past decade in elucidating the complex molecular mechanisms underlying AD, the translation of these findings into effective, implementable therapies is still incomplete [50]. Along with the inherent biological complexity of the disease [50], this translational gap is further compounded by insufficient preparedness of the healthcare system for patient identification, diagnosis, treatment delivery, and management of therapy-related AEs [68]. Accordingly, evaluating translational readiness extends beyond clinical efficacy alone and encompasses regulatory maturity [83], healthcare system readiness [84], manufacturing and scalability constraints, quality control and standardization requirements [85], long-term safety considerations, cost-effectiveness [66], and the overall feasibility of sustainable clinical integration into real-world clinical practice [84].

Given that AD requires long-term therapeutic management and continuous monitoring [62], the need for early diagnosis, specialized infrastructure, and long-term monitoring highlights the importance of evaluating the clinical, operational, and economic feasibility of therapeutic strategies [66,68,84]. The therapeutic strategies currently under evaluation represent markedly different stages of translational maturity, ranging from established first-line therapies to recently approved agents and early-stage investigational strategies. Accordingly, the evidence supporting these approaches also differs substantially, ranging from therapies supported by randomized clinical trials and regulatory approval to therapeutic strategies supported predominantly by preclinical evidence derived from in vitro and animal studies [66,85,86].

Symptomatic therapies have reached a high level of regulatory maturity. They are deeply integrated into clinical practice, supported by long-standing FDA approval and practical routes of administration, including oral and transdermal formulations. Routine cognitive monitoring is facilitated by widely implemented assessment tools, including the Mini-Mental State Examination. Although late-phase clinical trials have demonstrated consistent symptomatic benefits with AChEIs, these effects remain modest and transient, reflecting a low therapeutic ceiling [62]. Real-world evidence further indicates frequent early treatment discontinuation, largely due to limited tolerability and long-term efficacy [23]. Nevertheless, symptomatic therapies are relatively cost-effective for symptom management and constitute the mainstay of clinical support despite their inability to alter disease progression [86].

The regulatory approval of anti-amyloid mAbs, including lecanemab in 2023 and donanemab one year later, represents a major scientific and regulatory milestone following decades of AD research [83]. However, regulatory approval does not currently equate to full translational maturity [66,83]. While these agents achieve statistically significant efficacy, this clinical benefit translates into an estimated 6–12-month delay in cognitive decline in selected early-stage patients [66].

Their broader implementation is constrained by ARIA-related safety concerns, repeated intravenous infusions, MRI monitoring, biomarker-confirmed diagnosis, and the availability of specialized infrastructure and trained personnel required for longitudinal patient management [66,84]. Economic sustainability is also incompletely addressed. Current analyses suggest that mAbs are not cost-effective at their current annual price and may only become economically viable under conditions such as significant price reduction, shorter treatment duration with sustained benefit, or more selective administration in high-responder subpopulations [68].

In contrast to currently approved therapies, exosome-based strategies are still in the early stages of clinical translation [55], and no EV-based therapeutic product has yet to receive regulatory approval for clinical use [87]. A major translational barrier is the lack of standardized, scalable manufacturing processes, including harmonized protocols for exosome isolation, purification, characterization, cargo loading, and surface engineering [55,71,87].

Beyond manufacturing scalability, regulatory translation also requires robust quality assurance. Large-scale production under Good Manufacturing Practices (GMP), together with donor traceability, standardized release testing, batch consistency, and robust chain-of-custody procedures, is essential to ensure product reproducibility and clinical reliability [71,85,87]. Complementing these requirements, frameworks such as the Minimal Information for Studies of Extracellular Vesicles (MISEV) 2023 guidelines provide an important basis for methodological standardization and reproducibility across studies [60].

Despite this progress, the technically demanding exosome manufacturing, together with the need for specialized infrastructure and high production costs, continues to limit broad clinical deployment [60,87]. The implementation of optimized bioreactor systems for large-scale EV production may partially enhance clinical feasibility and enable more standardized, high-yield production of therapeutic exosomes [64].

Although short-term safety data are encouraging, comprehensive long-term safety evaluation is necessary before clinical implementation [71]. Following encouraging preclinical findings (see Section 3.1), clinical translation has entered the early-phase evaluation stage, beginning with the Phase I/II study by Wang et al. [48] (NCT04388982), which primarily focused on safety and tolerability. Cognitive outcomes and biomarker changes were evaluated as exploratory endpoints [48,55]. Beyond these considerations, successful integration of exosome-based therapies into clinical practice will additionally require strong operational rigor, ethical alignment, and coordinated collaboration among academic institutions, industry stakeholders, and regulatory authorities. Support from organizations such as the Alzheimer’s Association and the International Society for Extracellular Vesicles may further facilitate standardization efforts and improve translational progress within the field [85,88]. For ease of comparison, the overall findings across all five assessment parameters are summarized in Table 1.

To conclude, the three therapeutic platforms differ substantially in their translational maturity, regulatory readiness, and level of supporting evidence; therefore, their comparative evaluation should be interpreted within the context of their respective stages of clinical development. Symptomatic therapies demonstrate the highest degree of clinical readiness due to their established regulatory acceptance, sustainable clinical workflow, and relative cost-effectiveness, despite offering only modest and temporary symptomatic benefit. Anti-amyloid mAbs represent a critical step toward disease modification; however, their long-term feasibility is still under investigation due to limited efficacy, extensive monitoring requirements, safety concerns, and unresolved economic and infrastructural challenges. Exosome-based strategies, although currently characterized by low translational maturity, largely reflect the field’s immaturity rather than intrinsic technological limitations. Their long-term therapeutic potential is promising, and ongoing advances in engineering, manufacturing standardization, and clinical evaluation may largely reshape their future feasibility and real-world applicability in AD therapeutics.

## 4. Conclusions and Future Perspectives

The comparative analysis provided in this review emphasizes the ongoing evolution of AD treatment. While conventional strategies are the standard in current clinical practice, they also have important limitations, including modest clinical efficacy, safety concerns, and high costs, underlining the need for more advanced delivery platforms. The therapeutic landscape is heading towards the development of multi-targeted therapies that can alter the course of the disease.

The transition from purely symptomatic interventions to disease-modifying strategies is a significant milestone in AD therapy. However, the clinical benefit of anti-Aβ mAbs remains modest, largely due to limited BBB penetration and notable safety concerns. In contrast, exosomes may overcome several shortcomings of conventional strategies, particularly due to their favorable biocompatibility, low immunogenicity, efficient BBB translocation, and their prospect for multi-target engineering. These advantages support their conceptual ability to modulate amyloid clearance, neuroinflammation, and synaptic preservation, while addressing the multifactorial nature of AD pathobiology and supporting personalized therapeutic interventions.

Despite their conceptual advantages, the clinical application of exosome-based therapies is still in its infancy. Their successful translation into clinical practice will depend on overcoming key challenges related to manufacturing standardization, regulatory implementation, long-term safety validation, and economic feasibility. Therefore, effective clinical translation will require coordinated collaboration among academia, industry, regulatory authorities, and healthcare systems. Continued advances in bioengineering, manufacturing technologies, and well-designed clinical studies will be essential to establish the long-term therapeutic value of exosome-based interventions.

Overall, exosome-based strategies represent a possible solution to current translational gaps in AD therapy by enabling a transition toward precision medicine approaches and supporting targeted, safer, and potentially more effective interventions. The relatively low translational maturity of exosome-based therapies largely reflects their early validation stage and the incomplete understanding of exosome biology rather than an absence of therapeutic impact. In the long run, exosomes might not only support conventional treatments but could evolve into a safer and more effective alternative to current immunotherapies, as technological and clinical barriers are addressed. Ultimately, advances in bioengineering, methodological standardization, and robust clinical validation will determine whether exosomes can evolve into clinically viable therapeutic platforms for Alzheimer’s disease.

## Figures and Tables

**Table 1:** Comparative overview of conventional and exosome-based therapeutic strategies in AD across key evaluation parameters, including mechanistic targeting, delivery efficiency and blood–brain barrier penetration, safety and tolerability, therapeutic personalization, and translational readiness.

Evaluation Parameter	Symptomatic Treatments (AChEIs, NMDA Receptor Antagonists)	Disease-Modifying Therapies (anti-Aβ mAbs)	Exosome-Based Strategies
Mechanistic Targeting	Single-target neurotransmitter modulation; transient symptomatic benefit; no impact on core neurodegenerative mechanisms [50,52,53,54].	Amyloid-centric, single-pathway targeting; partial disease modification; limited efficacy against multifactorial pathology [50,51,52,53].	Designable multi-target platform; concurrent modulation of amyloid clearance, synaptic function, and neuroinflammation; combinatorial cargo delivery possible but design-dependent; contribution to pathophysiology context-dependent [36,55,56,57,58,59].
Delivery Efficiency and BBB Penetration	Small molecules with limited CNS bioavailability due to first-pass metabolism and systemic clearance; modest BBB penetration [18,53,61,62].	High target affinity but very low BBB penetration (~0.1%); intravenous delivery with safety-driven dose limitations [8,53].	Intrinsic BBB crossing via receptor-mediated transcytosis and fusion; surface engineering and intranasal delivery enhance brain targeting and reduce off-target exposure [18,53,60,63,64].
Safety and Tolerability	Well-characterized safety profile; mainly GI-related and cardiovascular AEs; generally suitable for long-term use [23,53,54,66].	Risk of ARIA requiring frequent MRI monitoring; higher risk in ApoE ε4 carriers; systemic and infusion-related AEs [8,53,54,66,67,68].	Favorable short-term safety and low immunogenicity in preclinical and early clinical studies; long-term toxicity, biodistribution, and immune effects remain insufficiently defined [30,55,70,71].
Therapeutic Personalization	Non-personalized by design; dose titration addresses tolerability, not disease biology [5,50,62,78,80,81].	Biomarker-based patient selection (PET/CSF, ApoE) supports safety stratification rather than response optimization [6,50,54,79].	High theoretical personalization via cargo selection, cellular origin, and surface modification; integration of multi-omics and AI could enable true patient-specific therapeutic design [21,50,64,73,75,76,82].
Translational Readiness	Highest translational maturity; supported by randomized clinical trials and FDA approval; cost-effective and fully integrated into routine care; low therapeutic ceiling (~6–12 months) [23,62,86].	Partial translational readiness despite regulatory approval and randomized clinical evidence; constrained by modest efficacy, safety monitoring burden, high cost, and infrastructure demands [66,68,83,84].	Low translational readiness; supported predominantly by preclinical in vitro and animal studies; limited early-phase clinical evidence; major barriers include lack of standardization, scalable GMP production, and regulatory clarity; long-term feasibility depends on manufacturing and regulatory advances rather than efficacy alone [55,60,64,71,85,88].

## Data Availability

No new data were created or analyzed in this study. Data sharing does not apply to this article.

## Abbreviations

The following abbreviations are used in this manuscript:

AChEIs	Acetylcholinesterase inhibitors
AD	Alzheimer’s Disease
ApoE	Apolipoprotein E
ARIA	Amyloid-related imaging abnormalities
ARIA-E	ARIA with edema
ARIA-H	ARIA with hemorrhage
Aβ	Amyloid-β
BBB	Blood–brain barrier
CNS	Central nervous system
CSF	Cerebrospinal fluid
EVs	Extracellular Vesicles
FDA	Food and Drug Administration
GMP	Good Manufacturing Practice
mAbs	Monoclonal antibodies
MCI	Mild cognitive impairment
MRI	Magnetic Resonance Imaging
MSC-Exos	Mesenchymal Stem Cell-derived Exosomes
NMDA	N-methyl-D-aspartate
PET	Positron Emission Tomography
PM	Precision (or Personalized) Medicine
RVG	Rabies Virus Glycoprotein
siRNA	Short interfering RNA
